# OncoCan: a liquid biopsy assay for cell-free DNA quantification in canine plasma to support cancer prognosis

**DOI:** 10.3389/fvets.2026.1768078

**Published:** 2026-02-23

**Authors:** Virginia Sánchez, Gal.la Farreny, Elena Martinez-Merlo, Sara Trancon, José Carlos Montañés, Laura Enriquez, Arantxa Aguirrebeña, Agustín Arasanz Duque, Antonia Noce

**Affiliations:** 1 Complutense Veterinary Teaching Hospital and Department of Animal Medicine and Surgery, Veterinary Medicine School, Complutense University of Madrid, Madrid, Spain; 2 Clinica Veterinaria Alameda, Madrid, Spain; 3 Omica Biomed, Barcelona, Spain; 4 Molecular Biology Core, Hospital Clínic of Barcelona, Barcelona, Spain

**Keywords:** cancer, cfDNA, dogs, liquid biopsy, prognosis

## Abstract

Early cancer detection remains a major challenge in veterinary medicine. Cell-free DNA (cfDNA), released into the bloodstream through apoptosis, necrosis, or circulating tumor cells, can be quantified non-invasively via liquid biopsy and is already established in human oncology. In this study, we evaluated OncoCan, a targeted plasma cfDNA assay, by analyzing samples from 83 dogs with various neoplasms and 47 healthy controls to assess diagnostic and prognostic utility. Wilcoxon rank-sum testing revealed significantly higher cfDNA concentrations in neoplastic versus healthy samples (*p* = 4.45e–07). ROC curve analysis demonstrated high accuracy for lymphomas/leukemias (AUC = 0.95) and moderate accuracy for carcinomas (AUC = 0.75), sarcomas (AUC = 0.76), and melanomas (AUC = 0.69). Stratification by histological grade and clinical stage further supported cfDNA’s predictive capability. Three practical thresholds were established: <50 pg/μL to distinguish healthy from neoplastic cases; ≥100 pg/μL as a “high positive” threshold indicating aggressive disease; and ≥300 pg/μL as a “very high positive” threshold strongly associated with systemic dissemination, high-grade histology, and poor survival. The <50 pg/μL cut-off showed robust diagnostic performance (AUC = 0.808, sensitivity = 82%, specificity = 73%), confirmed by survival analysis and hazard ratio modeling. These findings suggest that OncoCan provides a noninvasive, clinically applicable tool for cancer prognosis in dogs. Validation in larger cohorts is warranted to support its integration into routine veterinary oncology practice.

## 1 Introduction

Cancer is the most common cause of death among dogs, particularly in middle-aged and older animals. Lifetime prevalence estimates suggest that 25–40% of all dogs and ≈50% of dogs over 10 years will develop cancer, with malignant tumors occurring in approximately 30 per 1,000 dogs (1). The prognosis remains poor for many canine cancers due to late clinical detection; only ≈12% are diagnosed before symptom onset (2).

Early detection is therefore critical, yet it remains a persistent challenge in veterinary medicine. Conventional diagnostic workflows, including imaging, cytology, and histopathology, are typically initiated after clinical signs emerge and could require anesthesia or invasive procedures.

Blood-based biomarkers such as thymidine kinase-1 (TK1) and C-reactive protein (CRP) have demonstrated limited sensitivity and specificity (3, 4). Likewise, many classes of circulating biomarkers like proteins, enzymes, and cytokines has been reported to lack tumor specificity, frequently overlap with inflammatory conditions, and exhibit substantial biological and analytical variability that affects reproducibility and routine applicability (5–7). Even emerging biomarker classes, such as circulating nucleosomes, demonstrate variable performance and significant signal overlap between cancer and non-cancer states (8). These shared limitations underscore the need for alternative, more robust approaches, suitable for routine use, such as cfDNA-based assays.

Cell-free DNA (cfDNA) refers to extracellular, double-stranded DNA fragments released into the bloodstream via apoptosis, necrosis, and active secretion (9).

In health, cfDNA mainly reflects normal cellular turnover; in cancer, cfDNA concentrations can be markedly elevated due to tumor cell death and lysis. In human oncology, a substantial fraction of circulating cfDNA derives from tumor cells, carrying somatic mutations, copy-number variations (CNVs), and aberrant promoter methylation that recapitulate the primary tumor’s molecular landscape (10, 11). cfDNA thus enables early cancer detection, staging, monitoring of minimal residual disease, and assessment of therapeutic response (12, 13).

Liquid biopsy provides a well-established method for measuring cfDNA and other tumor-derived analytes in blood and various body fluids (e.g., cerebrospinal fluid, urine, pleural fluid, saliva). Compared to tissue biopsies, liquid biopsy is minimally invasive, carries low risk, and enables serial sampling to monitor tumor dynamics and heterogeneity, potentially capturing subclonal modifications across primary and metastatic sites (14, 15).

In canine oncology, cfDNA research is in its early stages but encouraging. Several studies report higher cfDNA concentrations or detectable circulating tumor DNA in dogs with mammary tumors, lymphomas, leukemias, and hemangiosarcomas compared with healthy controls (16). Notably, dogs with lymphoid neoplasia and plasma cfDNA exceeding a threshold (e.g., ~1,250 μg/L) showed poorer survival, suggesting a prognostic role for cfDNA levels (17). Shallow whole-genome sequencing and fragmentation/CNV analyses have demonstrated the feasibility of distinguishing tumor-bearing dogs from controls based on cfDNA patterns, supporting cfDNA’s diagnostic potential across canine cancers (18).

Despite these insights, evidence remains limited to a few tumor types, and comprehensive prospective validation across diverse canine cancers and prognostic endpoints is lacking. There is a critical need to translate cfDNA-based liquid biopsy into clinically robust, reproducible assays that veterinary clinicians can adopt for screening, diagnostic triage, and outcome prediction.

Here, we evaluate OncoCan, a targeted plasma cfDNA assay, for (i) diagnostic discrimination between dogs with confirmed neoplasia and healthy controls and (ii) prognostic association with survival in dogs with neoplasia. Using Wilcoxon rank-sum testing, receiver operating characteristic (ROC) analysis, and survival modeling, we assessed cfDNA concentrations (50–700 bp fragment range) in relation to histopathologic diagnosis and outcome. Our objectives were to define OncoCan’s diagnostic performance across multiple tumor types, establish clinically relevant cfDNA concentration thresholds, and lay the groundwork for integrating cfDNA-based liquid biopsy into routine veterinary oncology to enable earlier detection and dynamic disease monitoring.

## 2 Materials and methods

### 2.1 Study population

Between November 2023 and December 2024, a total of 130 canine patients were enrolled from the Complutense Veterinary Teaching Hospital (HCVC) and a private veterinary clinic (CV Alameda). Peripheral blood samples were collected from each patient as part of their routine diagnostic or pre-anesthetic protocol. A portion of each sample was used for research purposes, with informed consent obtained from the owners. No additional procedures were performed, thereby ensuring that animal welfare remained uncompromised.

All animals were classified into two groups based on their clinical status: a control group (CG) and an active neoplasia group (ANG).

The CG consisted of dogs attended by the Alameda veterinary center for elective procedures such as castration, sterilization, or dental cleaning. Inclusion criteria required the absence of clinical signs of disease and normal pre-surgical laboratory findings, with no indications of acute illness. Dogs undergoing treatment for non-oncological chronic conditions were also eligible, provided that both clinical and laboratory parameters confirmed disease stability.

Exclusion criteria for the CG included the presence of acute systemic illness, uncontrolled chronic disease, or any suspicion or confirmation of neoplastic processes during the pre-surgical evaluation. Data collected for this group included breed, age, sex, reproductive status, body weight, date of sample collection, fasting status at the time of sampling, prior clinical condition, and current treatments.

The ANG included dogs evaluated at the oncology consultation with clinical signs of neoplastic disease confirmed by cytology and/or histopathology. Only dogs that had not received any form of cancer treatment were included in the study. Exclusion criteria included residual diseases that are not clinically evident, previous local treatments (surgery, radiotherapy, electrochemotherapy), systemic cancer therapies, and corticosteroid treatment in cases of lymphoma or leukemia.

Tumors in the ANG were classified according to three integrated criteria: type of neoplasia (carcinomas, lymphomas/leukemias, mast cell tumors, melanomas, sarcomas, other round cell tumors, and benign tumors); clinical stage (localized or disseminated disease) and tumor histopathological grade. Clinical stage was determined through imaging and laboratory studies, based on an adaptation of the World Health Organization TNM staging system for each tumor type. For solid tumors, disseminated disease was defined as the presence of regional and/or distant metastasis (i.e., any TNM stage other than N0M0). All cases of lymphoma/leukemia were considered disseminated regardless of clinical stage. Regarding tumor grade (high and Low) was primarily based on histopathological reports (19), with cytological criteria accepted for mast cell tumors (20) and lymphomas. All metastatic tumors were considered to exhibit high-grade malignant behavior, regardless of their cytological or histological grade, as they demonstrated marked aggressiveness. Data collected for the ANG included breed, age, sex, reproductive status, body weight, date of sample collection, fasting status at the time of sampling, tumor type, date of oncological diagnosis, diagnostic method, TNM-based staging, and tumor grade.

### 2.2 Sample collection and processing

A minimum of 5 mL of whole blood was collected via jugular or cephalic venipuncture into EDTA tubes, with the aim of obtaining approximately 2 mL of plasma after processing. Plasma was separated by centrifugation at 2,000×*g* for 10–15 min at room temperature. The resulting supernatant (2 mL) was initially stored at −40 °C and subsequently shipped to OMICA’s laboratory on dry ice. Transport conditions were maintained on dry ice, and delivery occurred within 24 h. Upon arrival, samples were stored at −80 °C until further processing (from a minimum of 2 days to a maximum of 2 weeks). The time interval between blood collection and plasma storage was carefully monitored to minimize cfDNA degradation caused by DNases present in the plasma.

For cfDNA preparation, plasma samples were thawed at room temperature for 20 min and subjected to a second centrifugation at 16,000×*g* for 4 min to remove residual cellular debris. Purified supernatants (2 mL plasma input per sample) were used for cfDNA extraction. cfDNA was isolated using the column-based NucleoSnap DNA Plasma kit (Macherey-Nagel, Germany) with vacuum processing on a NucleoVac 24 manifold (Macherey-Nagel, Germany). DNA was eluted once in 50 μL of ultrapure water (LabKem, Spain). The concentration and fragment size distribution of cfDNA were assessed using capillary electrophoresis on a TapeStation 4150 Bioanalyzer (Agilent Technologies, USA) with the High Sensitivity Cell-free DNA ScreenTape assay. A fragment size of 50–700 bp was selected to quantify cfDNA and to identify samples with high-molecular-weight (HMW) genomic DNA contamination. The 50–700 bp range refers to an analytical window applied during electropherogram interpretation and does not involve any physical size-selection or purification of cfDNA fragments. Samples showing a predominant HMW peak (>700 bp) or a marked shift toward large genomic fragments, indicative of leukocyte lysis or inadequate pre-analytical handling, were excluded from downstream analysis. This size-based quality criterion ensured that only samples enriched for true circulating cfDNA were included.

### 2.3 Statistical analysis

#### 2.3.1 Wilcoxon rank-sum tests

To assess the diagnostic potential of circulating cfDNA, we compared the cfDNA concentrations between healthy and tumor samples using Wilcoxon rank-sum tests, which were performed with an R custom script. To eliminate contamination from degraded genomic DNA, only samples with a minimum of 10% cfDNA fragments in the 50–700 bp range were included in the analysis. These comparisons allowed us to identify tumor types with significantly elevated cfDNA levels, suggesting biological relevance beyond its role as a quality control metric.

#### 2.3.2 Receiver operating characteristic (ROC) curve analysis

To identify good thresholds cut-off of cfDNA concentration able to distinguish healthy samples from neoplastic, we performed Receiver Operating Characteristic (ROC) curve analysis using the pROC package in R (21).

The ROC analysis is a widely used statistical method in biomedical research for evaluating the performance of classification models and biomarkers. It plots the true positive rate (TPR) indicative of sensitivity against the false positive rate (FPR) indicative of 1-specificity across a range of threshold values. In this way, it allow the identification of cutoff points that best differentiate between clinical outcomes, such as disease presence or progression (22).

We first defined four ranges of cfDNA concentration based on arbitrary cut-offs (<50 pg/μL, 50–99 pg/μL, 100–299 pg/μL, ≥300 pg/μL) to explore the distribution of the cfDNA concentration in our samples. Tumor types of groups with samples representation across all ranges and a sufficient population size were selected for subsequent analyses.

ROC curve analysis was then performed to compare these tumor samples with healthy controls, allowing estimation of sensitivity and specificity for the optimal threshold identified in each group. In addition, ROC analyses were conducted to compare dogs with healthy versus low histological tumor grade as well as low versus high grade and localized versus disseminated disease, further assessing the predictive capability of cfDNA concentrations.

Given the number of animals included in the study, it was considered necessary to simplify the grading schemes to obtain more meaningful results; therefore, a two-tier grading system was applied whenever possible (e.g., the Kiupel classification for mast cell tumors). For tumor types graded on a three-tier system, low- and intermediate-grade tumors were grouped as low grade, distinguishing only high-grade tumors. Studies including a larger number of patients would be advisable to independently assess the performance of the technique in intermediate-grade tumors.

#### 2.3.3 Survival analysis

Kaplan–Meier survival analysis (23) was employed to evaluate time-to-event outcomes across the cohort, based on information provided by the veterinary clinics regarding the time elapsed from the diagnosis of the neoplastic process to either death due to the tumor or the date of the last known follow-up visit. This endpoint was used to calculate survival curves.

Patients received either local treatment (surgery, electrochemotherapy, or radiotherapy) or systemic therapy (chemotherapy, anti-angiogenic agents, or targeted therapies) based on therapeutic protocols established by tumor type, histological grade, and clinical stage. Survival times were calculated after the administration of these treatments, which have a substantial impact on clinical outcomes. The aim of this part of the study was to determine whether cfDNA levels could serve as an additional prognostic factor, beyond the intrinsic characteristics of each tumor and the therapeutic interventions applied, to help estimate survival times.

For survival analysis, samples were stratified into four categories: <50 pg/μL, 50–100 pg/μL, 100–300 pg/μL and >300 pg/μL. Patient deaths were coded as events (1), while individuals alive or lost to follow-up were censored (0), ensuring a right-censored approach. Kaplan–Meier estimation is widely used in clinical research to compare survival distributions between groups. Survival curves were generated using the R survival v3.2–7 package (24), and visualizations were produced with the survminer R package (25) enhanced plotting features and statistical annotations.

The follow-up period extended from the diagnosis of the neoplastic process to the date of manuscript preparation, with a maximum duration of 24 months for most patients. In three cases, survival time was calculated from the initial date of diagnosis, although cfDNA quantification was performed on untreated recurrences and/or metastases. A total of 49.4% of patients died because of the tumor (in most cases, euthanasia was performed after staging tests confirmed tumor progression and irreversible deterioration of quality of life; confirmation of cause of death by necropsy was carried out in fewer than 20% of patients). At the time of manuscript preparation, 28.9% of patients were alive, with tumor stabilization and/or good quality of life. Loss to follow-up due to lack of owner response accounted for 21.7% of the cases.

#### 2.3.4 Hazard ratio analysis

Hazard ratio analysis was performed using the cox proportional hazards model implemented in the survival package v3.2–7 (24) in R. cfDNA concentration was categorized into four ranges: <50 pg/μL, 50–100 pg/μL, 100–300 pg/μL and >300 pg/μL. Survival time was defined as the interval from the date of neoplasia diagnosis to death due to the tumor or last follow-up. To ensure a right-censored survival analysis, patient deaths were coded as events (1), while individuals alive or lost to follow-up were censored (0). The model estimated hazard ratios (HR) and 95% confidence intervals (CI) for each cfDNA category. Statistical significance was assessed using Wald tests, and global model fit was evaluated with the log-rank test. Model performance was summarized by the Akaike information criterion (AIC) and concordance index (C-index). Visualization of HR estimates and confidence intervals was generated using the survminer package (25).

## 3 Results

A total of 130 dogs met the inclusion criteria. The CG comprised 47 dogs from 31 breeds, aged 2–15 years. The ANG comprised 83 dogs from 44 breeds, aged 3–17 years.

Demographic characteristics of all enrolled dogs, together with tumor type, stage, and grade for the ANG, are summarized in Table 1 and fully reported in Supplementary Table S1.

**Table 1:** Summary of data collected for dogs included in the control group (CG) and active neoplasia group (ANG).

Variable	Category	CG	ANG
Patients (N)	Canine	47	83
Age at diagnosis (years)	Mean ± SD (Range)	8.9 ± 3.8 (range 2–15)	12.1 ± 2.8 (range 3–17)
Weight (kg)	Mean ± SD (Range)	17.5 ± 13.0 (range 2–62)	21.6 ± 12.0 (range 3–60)
Sex (N)	Male	23	37
	Female	24	46
Reproductive status (%)	Neutered/spayed	31	63
Breeds (N)	Types	31	44
Tumor type (N)	Benign	–	4
	Carcinomas	–	26
	Lymphoma/Leukemia	–	20
	Mast cell tumor (Mastocytomas)	–	12
	Melanomas	–	7
	Round cell tumors	–	2
	Sarcomas	–	12
Clinical staging (N)	Disseminated	–	39
	Localized	–	43
Histological grade (N)	High	–	54
	Low	–	22
	Unclassifiable	–	7

### 3.1 cfDNA percentage shows tumor-specific elevation

**Figure 1:**
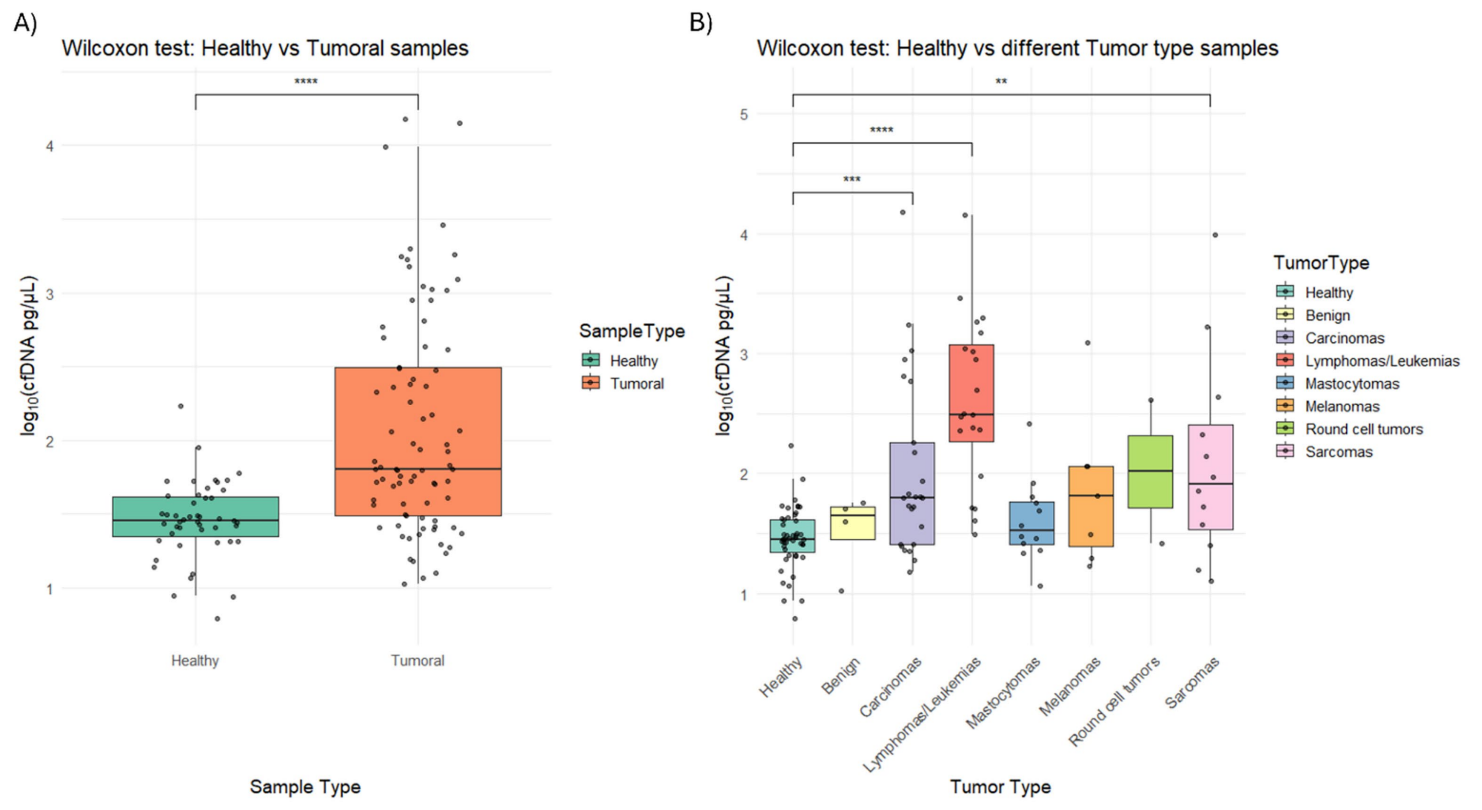
**(A)** Box plot comparing cfDNA concentration (log-transformed, 500–700 bp range) between healthy and tumor samples, assessed using the Wilcoxon test [statistical significance is indicated by stars: *p* < 0.05 (*), <0.01 (**), <0.001 (***), <0.0001 (****)]. **(B)** Box plot showing cfDNA concentration across healthy samples and different tumor types (benign, carcinomas, lymphomas/leukemias, mast cell tumors aka mastocytomas, melanomas, round cell tumors, sarcomas). Statistical significance was evaluated using the Wilcoxon test.

Statistically significant differences were obtained from the comparison of Wilcoxon rank-sum tests of cfDNA concentration across healthy controls and neoplastic samples (*p*-value = 4.45e-07) (Figure 1A; Supplementary Table S2A). When we compared healthy controls with each tumor type, lymphomas/leukemias showed the highest significance (*p*-value = 7.08e-9), followed by carcinomas (*p*-value = 5.06e-4), and sarcomas (*p*-value = 6.88e-3) (Figure 1B; Supplementary Table S2A).

### 3.2 Diagnostic utility of cfDNA concentration level (50–700 bp fragments range) and discriminatory power for histological grade and clinical stage

**Figure 2:**
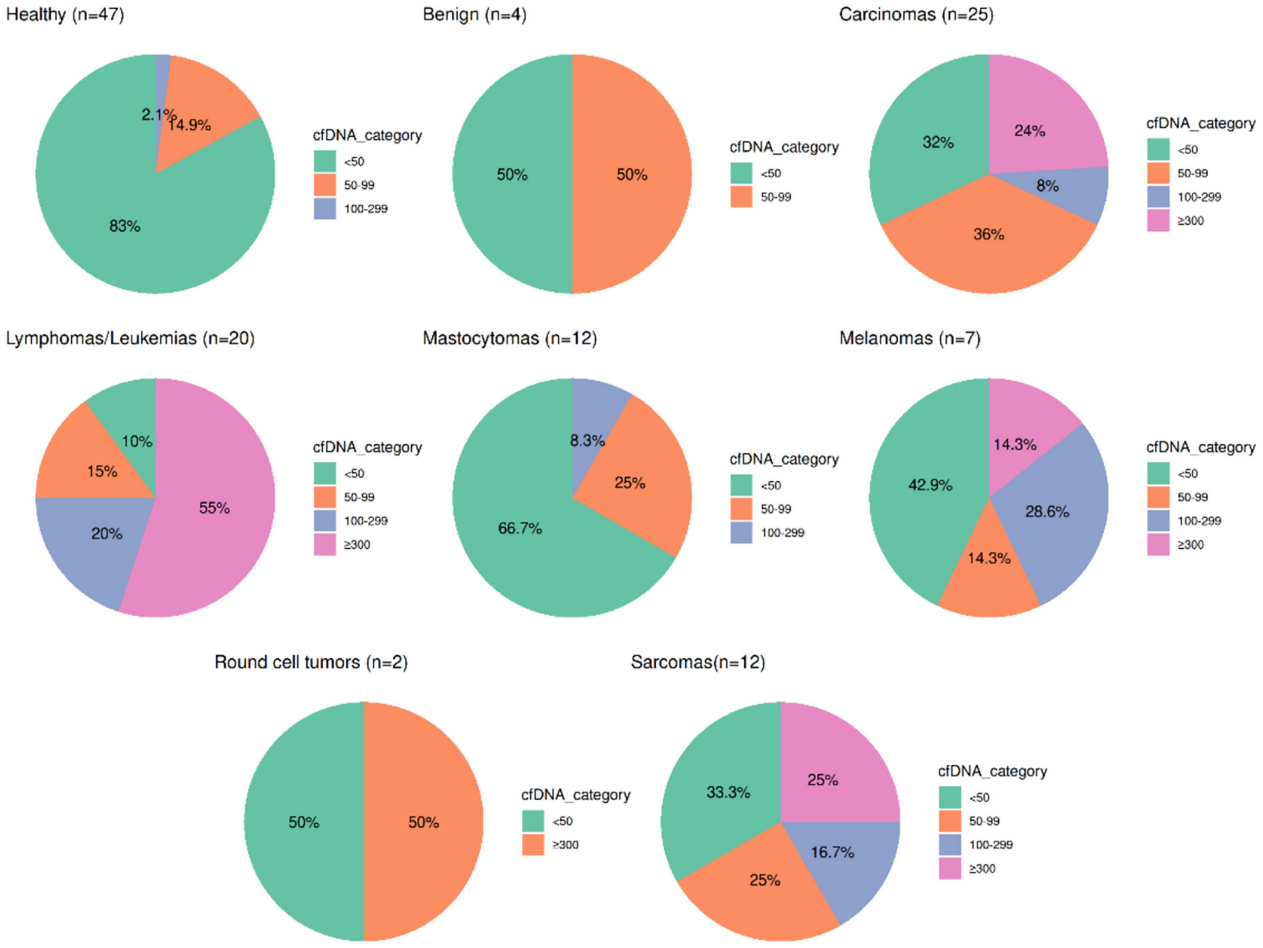
Distribution of cfDNA concentration (50–700 bp fragment range) across healthy and neoplastic sample groups analyzed in this study. Each pie chart represents one group, with cfDNA categorized into four ranges: <50 pg/μL, 50–99 pg/μL, 100–299 pg/μL, and >300 pg/μL (as indicated in the legend). Carcinomas, lymphomas/leukemias, melanomas, and sarcomas were the only tumor types with sample representation across all cfDNA concentration ranges and were therefore selected for subsequent analyses.

Analysis of cfDNA concentration (50–700 bp fragments range) revealed distinct patterns between healthy and neoplastic samples. CG samples predominantly exhibited low cfDNA levels, with 83% below 50 pg/μL. In contrast, ANG showed higher cfDNA concentrations and greater variability. Lymphomas/leukemias had only 10% of samples below 50 pg/μL, while 55% exceeded 300 pg/μL; the remaining samples were distributed across intermediate ranges (20% in 100–299 pg/μL and 15% in 50–99 pg/μL). Carcinomas, melanomas, and sarcomas displayed heterogeneous distributions, with representation across all cfDNA ranges. Benign tumors, mast cell tumors (aka mastocytomas), and round cell tumors retained a high proportion of samples below 50 pg/μL, partly due to smaller sample sizes (Figure 2).

To validate the discriminatory power of cfDNA concentration level, ROC curve analysis was performed on the tumor groups that included samples spanning all cfDNA concentration ranges defined for dataset exploration (lymphomas/leukemias, carcinomas, melanomas, sarcomas) which included 64 total samples (Supplementary Table S3). We next performed ROC analysis for all these tumors groups combined to identify a more generalized threshold.

**Figure 3:**
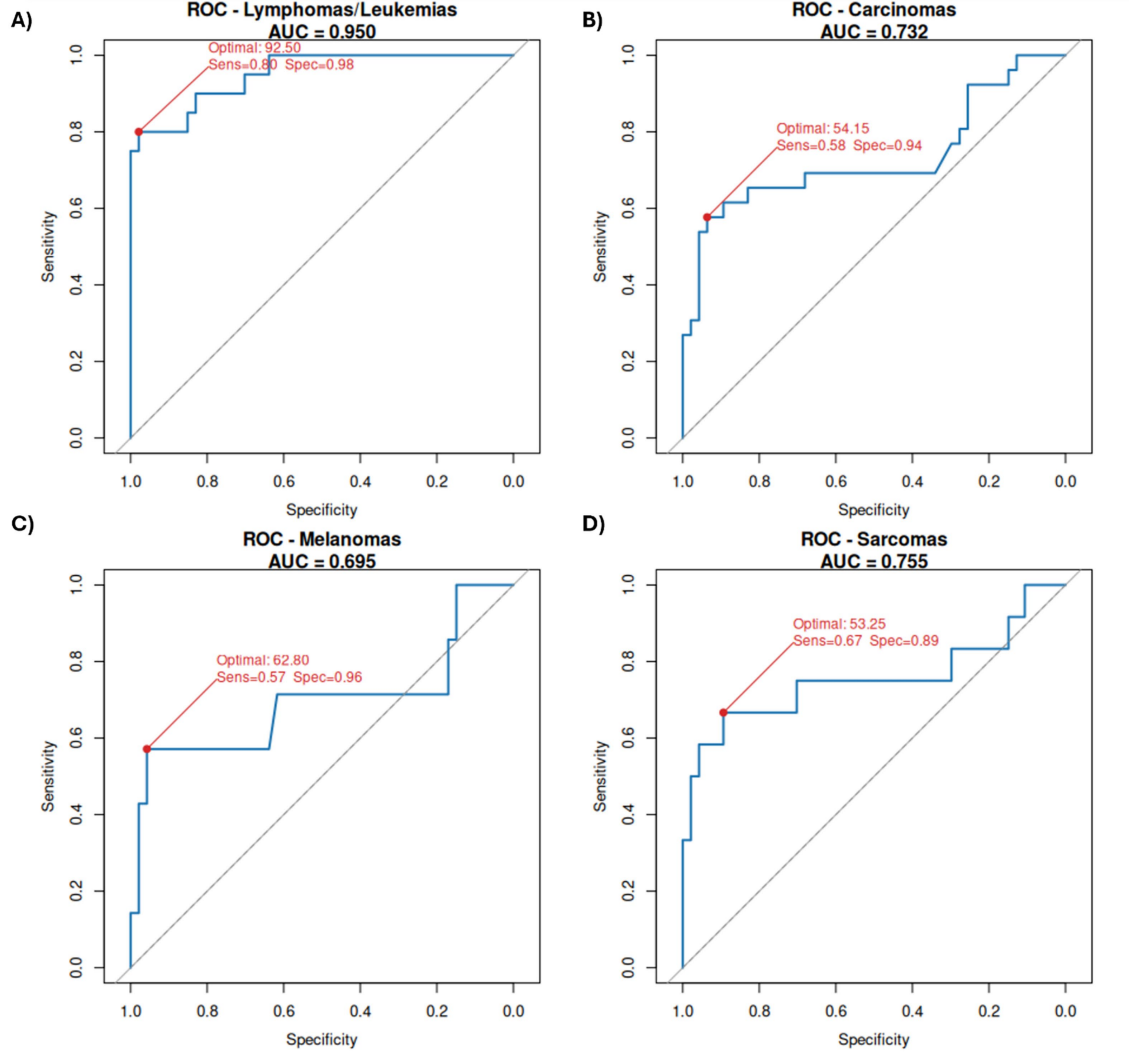
ROC curves illustrating the performance of cfDNA concentration (50–700 bp fragment range) as a classifier for the four most representative tumor types analyzed in this study [Lymphomas/Leukemias **(A)**, Carcinomas **(B)**, Melanomas **(C)**, and Sarcomas **(D)**] compared to healthy controls. The area under the curve (AUC) quantifies the trade-off between true positive rate (sensitivity) and false positive rate (1 – specificity), indicating the discriminatory power of cfDNA concentration for each tumor type, reference diagonal line (AUC = 0.5). The optimal threshold, sensibility and specificity values derived from the analysis are indicated on each plot.

**Figure 4:**
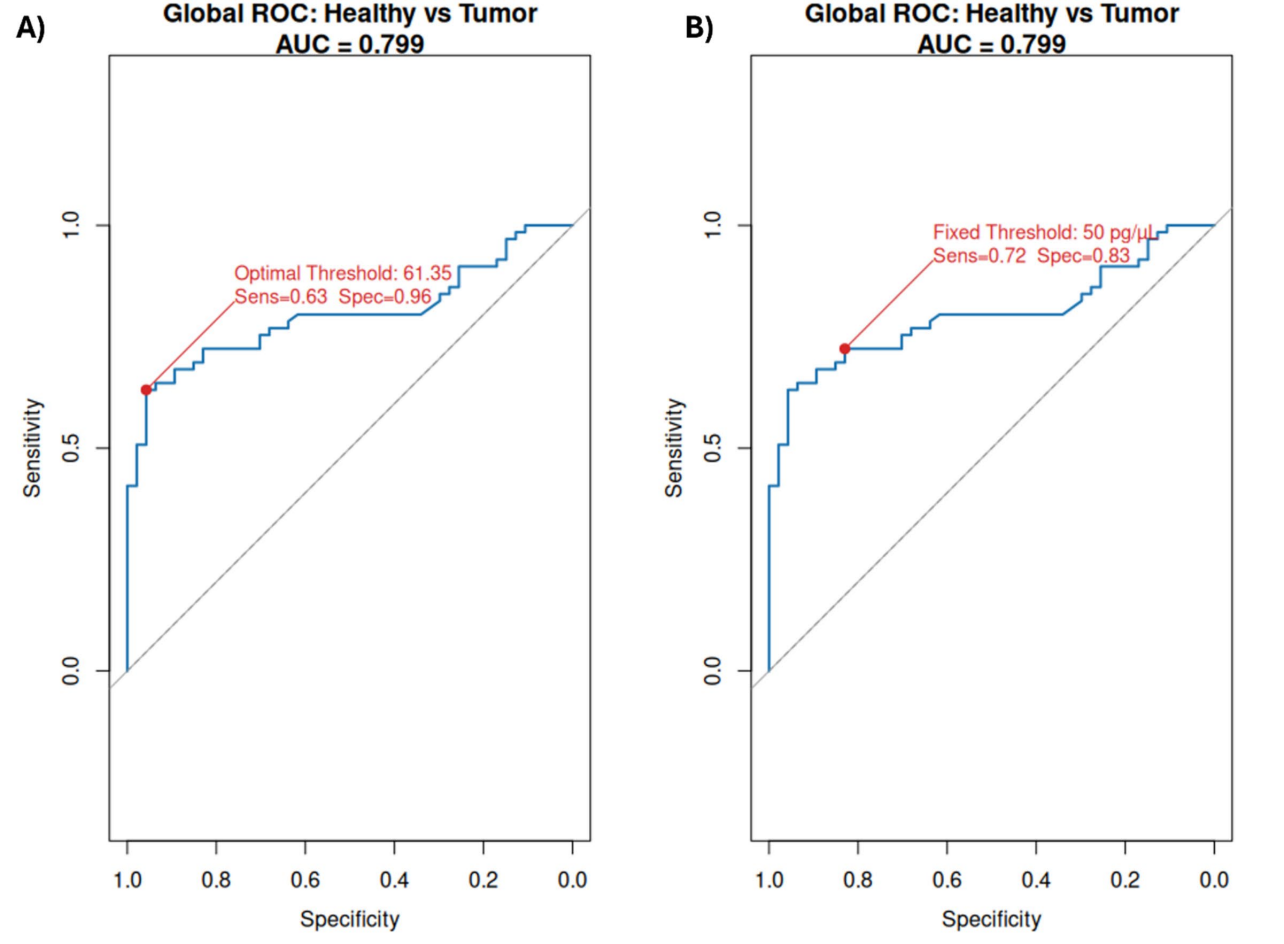
**(A)** ROC curves illustrating the performance of cfDNA concentration (50–700 bp fragment range) as a classifier between healthy and active neoplasia samples independent of tumor type (tumors groups of Lymphomas/Leukemias, Sarcomas, Melanomas, and Carcinomas combined). **(B)** ROC curves were obtained using the same samples and a fixed threshold of 50 pg/μL cfDNA concentration. The area under the curve (AUC) quantifies the trade-off between true positive rate (sensitivity) and false positive rate (1 – specificity), indicating the discriminatory power of cfDNA concentration for each tumor type, reference diagonal line (AUC = 0.5). The optimal threshold, sensibility, and specificity values derived from the analysis are indicated on each plot.

Lymphomas/leukemias achieved the highest accuracy (AUC = 0.95) with an optimal threshold of 92.5 pg/μL, yielding 80% sensitivity and 97.9% specificity. Carcinomas showed moderate performance (AUC = 0.73, threshold = 54.15 pg/μL, sensitivity = 58%, specificity = 94%). Sarcomas had similar discrimination (AUC = 0.75, threshold = 53.25 pg/μL, sensitivity = 67%, specificity = 89%). Melanomas presented the lowest AUC (0.69) with threshold = 62.8 pg/μL, sensitivity = 57%, specificity = 96% (Figure 3; Supplementary Table S2B). Combining all tumors the optimal threshold was 61.35 pg/μL with a 96% sensitivity and a 63% specificity (Figure 4A; Supplementary Table S2B). After evaluating these results, we considered that a minimal threshold of 50 pg/μL was a good cut-off for discrimination between healthy and active neoplasia samples, able to include several types of tumors, for this reason we tested it with a ROC curve analysis. Results showed slightly reduced sensitivity (83%) but an improved specificity (72%) for our dataset (Figure 4B; Supplementary Table S2B).

ROC curve analysis was also performed using the histological grade and cytological stage information provided by the veterinary clinic to assess the discriminatory power of cfDNA concentration for these classifications.

**Figure 5:**
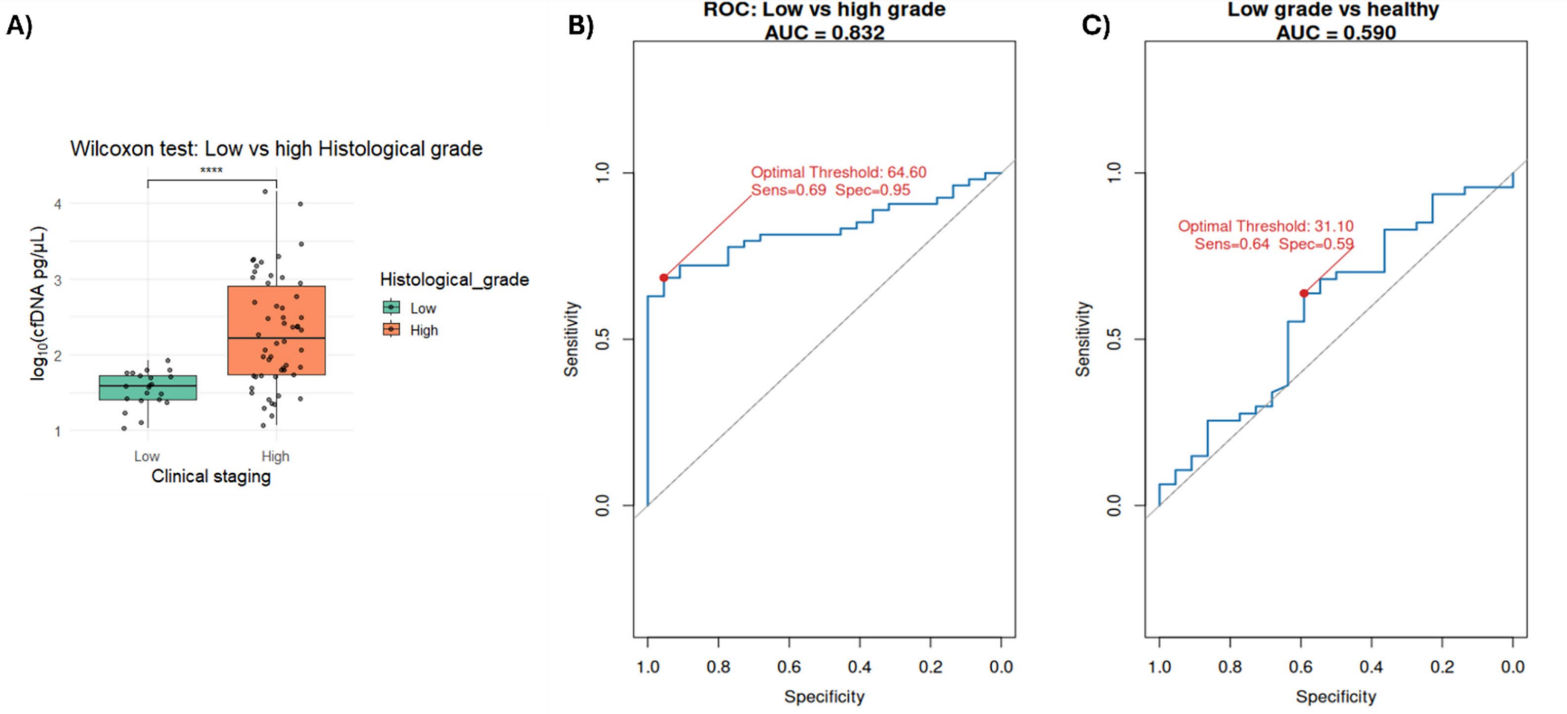
**(A)** Bar plot showing the distribution of samples by histological grade (high vs. low), as reported by the veterinary clinic for the ANG. **(B)** ROC curve evaluating the performance of cfDNA concentration (50–700 bp fragment range) as a classifier for high vs. low histological grade. **(C)** ROC curve evaluating the performance of cfDNA concentration (50–700 bp fragment range) as a classifier for low histological grade vs. healthy samples. The area under the curve reflects the trade-off between true positive rate (sensitivity) and false positive rate (1 – specificity), demonstrating the discriminatory power of cfDNA concentration, reference diagonal line (AUC = 0.5). The optimal threshold, sensibility, and specificity values derived from the analysis are indicated on the plot.

**Figure 6:**
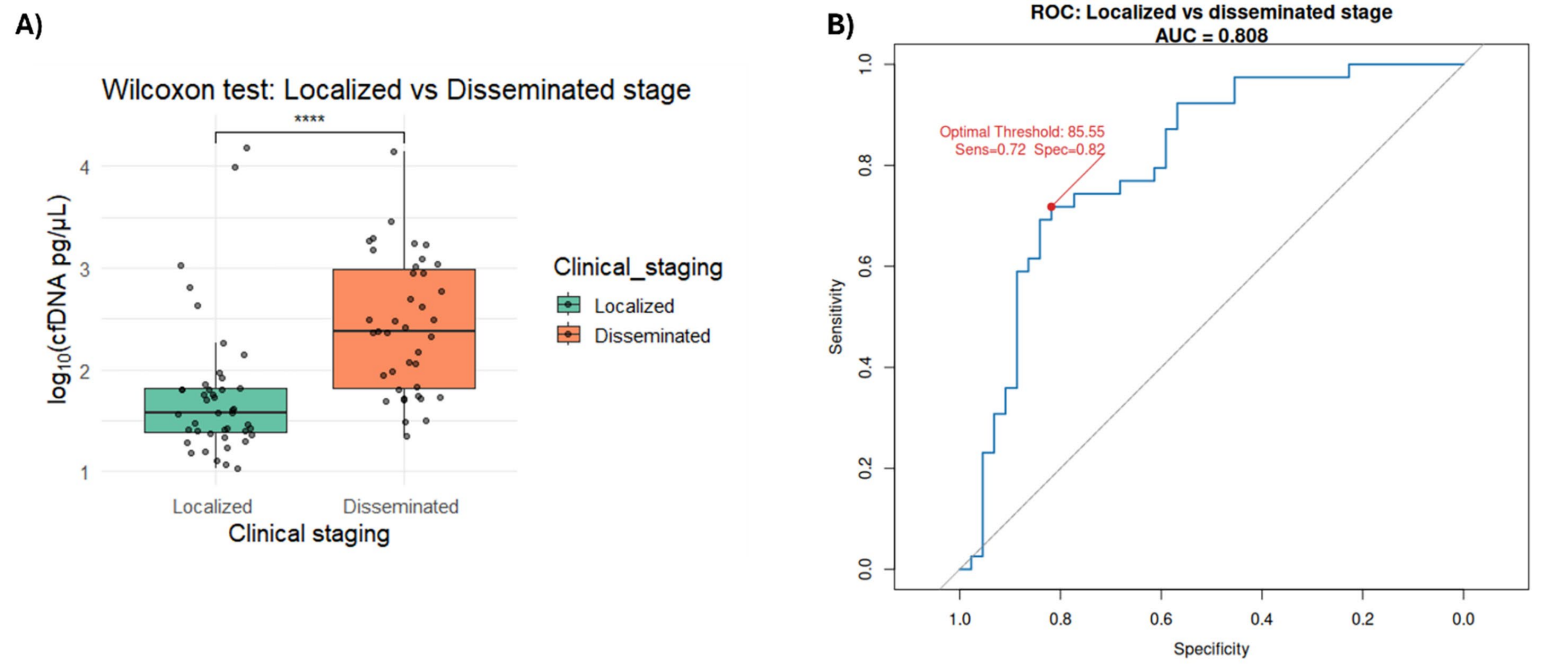
**(A)** Bar plot showing the distribution of samples by clinical staging—localized and disseminated—for each tumor type group. **(B)** ROC curve evaluating the performance of cfDNA concentration (500–700 bp range) as a classifier for localized vs. disseminated tumors. The area under the curve (AUC = 0.804) reflects the trade-off between true positive rate (sensitivity) and false positive rate (1 – specificity), demonstrating the discriminatory power of cfDNA concentration, reference diagonal line (AUC = 0.5). The optimal threshold, sensibility, and specificity values derived from the analysis are indicated on the plot.

For histological grade, we considered comparing healthy versus low grade as well as low versus high grade to assess both early and late-stage predictive capability of cfDNA concentrations. ROC analysis for low versus high grade yielded an AUC of 0.83, with an optimal threshold of 64.6 pg/μL (sensitivity 69%, specificity 95%) (Figure 5B; Supplementary Table S2B). In contrast, comparison of low-grade tumors versus healthy controls resulted in an AUC of 0.59, with an optimal threshold of 31.1 pg/μL (sensitivity 64%, specificity 59%) (Figure 5C; Supplementary Table S2B). It is important to note that our dataset contains substantially fewer low-grade cases (*N* = 22) compared with high-grade cases (*N* = 53), which limits statistical power for early-stage discrimination. To further characterize performance at the 31.1 pg/μL threshold, we generated a confusion matrix (Supplementary Table S2C), which yielded a sensitivity of 0.59 (95% CI: 0.36–0.79), specificity of 0.64 (95% CI: 0.49–0.77), and an overall accuracy of 0.62. For tumor stage (Localized vs. Disseminated), performance was slightly lower, with an AUC of 0.80, an optimal threshold of 85.55 pg/μL, sensitivity of 72%, and specificity of 82% (Figure 6; Supplementary Table S2B).

### 3.3 Survival analysis and hazard ratio

**Figure 7:**
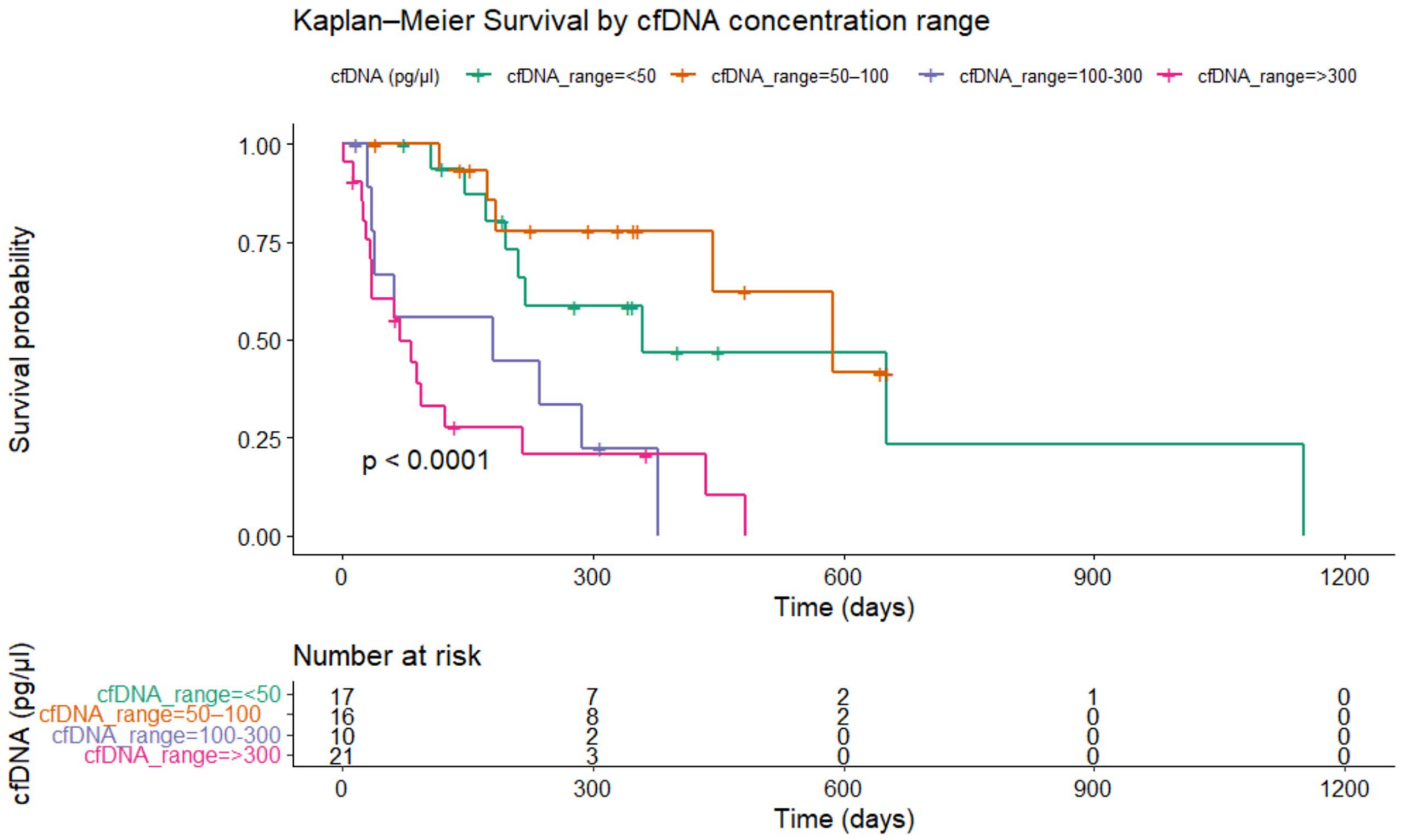
Kaplan–Meier curves stratified by cfDNA concentration ranges (<50, 150–100, 100–300, >300 pg/μL) in samples from lymphomas/leukemias, carcinomas, melanomas, and sarcomas tumor groups. Survival time was defined from the date of neoplasia diagnosis to death or last follow-up for 64 dogs, with 39 deaths coded as events (1), and 25 dogs either alive or lost to follow-up censored (0).

**Figure 8:**
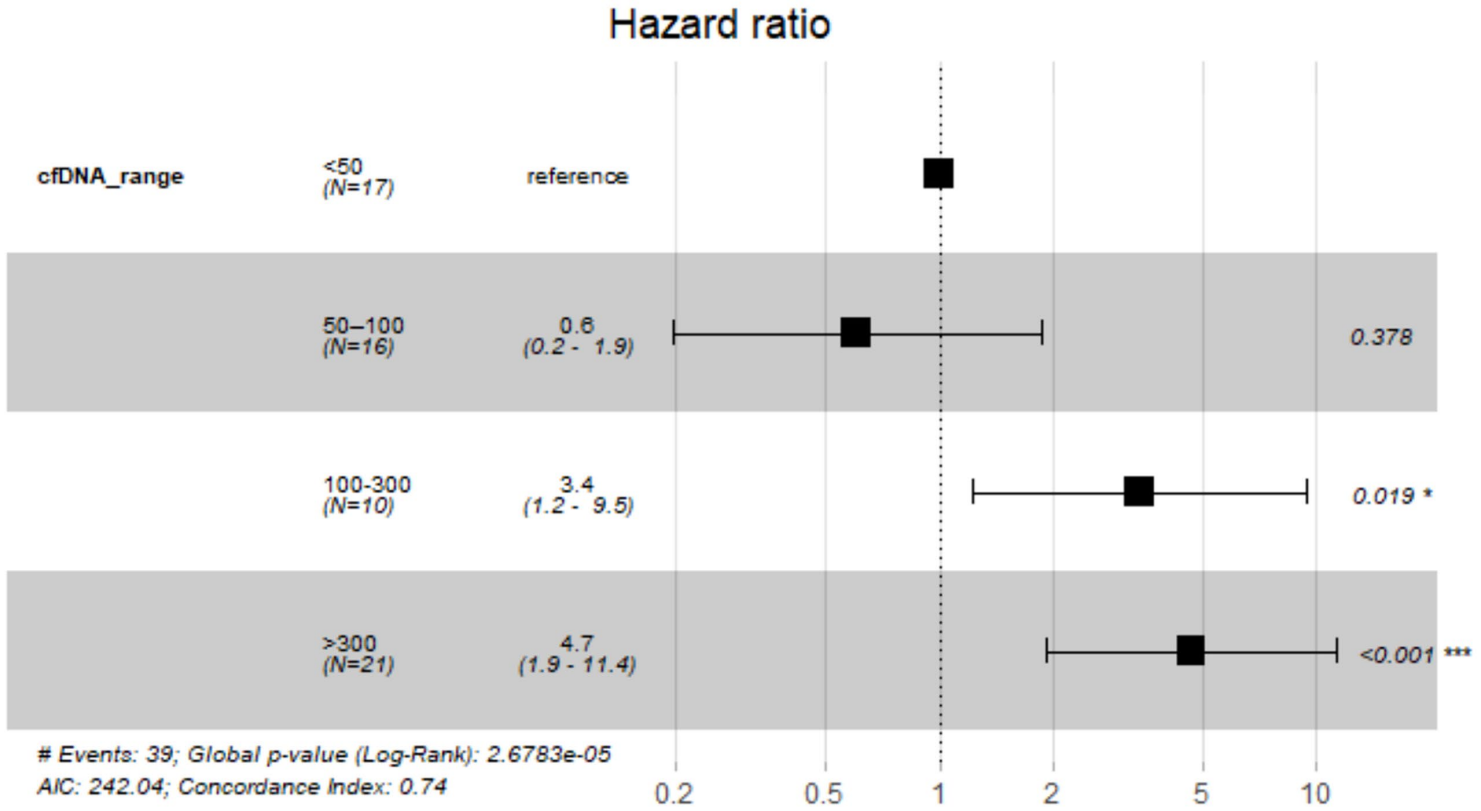
Forest plot of hazard ratios for cfDNA concentration ranges in a total of 64 samples from lymphomas/leukemias, carcinomas, melanomas, and sarcomas tumor groups, 39 of them deaths and coded as events (1). The Cox proportional hazards model compared four cfDNA categories: <50 pg/μL (reference), 50–100 pg/μL, 100–300 pg/μL, and >300 pg/μL.

Kaplan–Meier analysis revealed significant differences in survival across cfDNA concentration ranges (log-rank *p* < 0.0001) Dogs with cfDNA 50–100 pg/μL showed survival curves closely aligned with the <50 pg/μL group, with relatively slow decline over time. In contrast, dogs with cfDNA 100–300 pg/μL exhibited markedly reduced survival probability, with a sharp drop during the first 300 days. The poorest outcomes were observed in the >300 pg/μL group, which showed the steepest early decline and the shortest overall survival. Survival time was defined from the date of neoplasia diagnosis to death or last follow-up for 64 dogs, with 39 deaths coded as events (1), and 25 dogs either alive or lost to follow-up coded as censored (0), ensuring a right-censored analysis (Figure 7; Supplementary Table S3A). Hazard ratio modeling confirmed the survival trend (Figure 8). Compared to the reference group (<50 pg/μL), patients with 50–100 pg/μL showed no significant difference in mortality risk (HR = 0.8, 95% CI: 0.2–1.9, *p* = 0.378). In contrast, cfDNA levels between 100 and 300 pg/μL were associated with a substantially increased risk of death (HR = 3.4, 95% CI: 1.2–9.5, *p* = 0.019). The highest cfDNA category, >300 pg/μL, demonstrated the strongest association with mortality, corresponding to nearly a fivefold increased risk (HR = 4.7, 95% CI: 1.9–11.4, *p* < 0.001). The global model fit was supported by the log-rank test (*p* = 2.68 × 10^−5^), with a concordance index of 0.74, indicating robust predictive performance (Figure 8; Supplementary Table S3B).

## 4 Discussion

This study evaluates OncoCan, a targeted plasma cfDNA assay, demonstrating its dual potential as both a diagnostic and prognostic tool in canine oncology. Our findings align with emerging evidence indicating that cell-free DNA (cfDNA) is elevated in dogs with neoplasia and correlates with tumor burden and progression.

We observed significantly higher cfDNA concentrations in tumor-bearing dogs compared to healthy controls, a pattern consistent with earlier reports. For example, (16) found elevated plasma cfDNA in dogs with malignant tumors, including lymphoma, leukemia, and metastatic cancers, and noted correlations between cfDNA levels, tumor stage, and clinical progression. Similarly, (17) reported that dogs with cfDNA above a defined threshold had significantly reduced survival, and that cfDNA levels reflected treatment response, particularly in lymphoma.

A critical methodological distinction in our study is the targeted measurement of cfDNA fragments within the 50–700 bp range using the Agilent TapeStation 4150 Bioanalyzer with the High Sensitivity Cell-free DNA ScreenTape assay. This approach was deliberately chosen over fluorometric quantification methods such as Qubit, which measure total double-stranded DNA indiscriminately, including high-molecular-weight (HMW) genomic DNA from cell lysis. Fragment size differentiation is essential because shorter cfDNA fragments (50–700 bp) are characteristic of apoptotic and necrotic tumor-derived DNA, whereas larger fragments often result from sample handling or contamination. Typically, cfDNA profiles show a dominant peak around 170 bp, corresponding to mononucleosomal fragments, and may include additional peaks from nucleosomal multimers. Fragments >700 bp are considered HMW DNA and excluded from the cfDNA region of interest. In this way it is possible to provide a percentage of cfDNA quality metric that quantifies contamination from HMW DNA and ensures accurate representation of biologically relevant cfDNA (Agilent Technologies, 2018). This methodological rigor strengthens the diagnostic performance of OncoCan by focusing on biologically meaningful cfDNA fragments.

Previous studies in human oncology have demonstrated that fragment size profiling enhances sensitivity for detecting tumor-derived cfDNA and reduces false positives from non-tumor DNA. For example, (26) showed that selecting cfDNA fragments between 90 and 150 bp, led to over two-fold enrichment of circulating tumor DNA and improved cancer detection across multiple cancer types, achieving AUCs > 0.99 for certain cancers. Additionally, size selection approaches, such as the sequencing of shorter cfDNA fragments in non-invasive prenatal testing, have been shown to decrease erroneous results by excluding large non-target DNA fragments, a principle directly applicable to oncology cfDNA analysis (27). Applying this principle in veterinary oncology provides a more accurate foundation for liquid biopsy-based diagnostics and prognostics.

Our ROC analysis further confirms cfDNA concentration as a robust diagnostic classifier, especially for lymphomas/leukemias (AUC = 0.95, sensitivity = 80% and specificity = 98%). This is in line with clinical validation studies of multi-cancer liquid biopsy assays in dogs: the large-scale CANDiD study (*n* > 1,100) reported cfDNA quantification combined with genomic profiling achieved a specificity of ~98.5% and sensitivities between 54 and 71% across cancer types. This strong performance of OncoCan in hematologic malignancies is comparable with similar liquid biopsy technologies, such as OncoK9™,^1^ which detected lymphoma, hemangiosarcoma, and osteosarcoma with ~85% sensitivity (28). ROC curve analysis identified optimal thresholds for distinguishing health from neoplastic cases across the four most representative tumor types in our dataset: 92.5 pg/μL for lymphomas/leukemias, 54.15 pg/μL for carcinomas, 62.8 pg/μL for melanomas, and 53.25 pg/μL for sarcomas. These values reflect the best trade-off between sensitivity and specificity for each tumor type.

To facilitate clinical interpretation, we proposed three practical cut-off points: <50 pg/μL as a diagnostic threshold to separate healthy from neoplastic cases, ≥100 pg/μL as a “high positive” indicator of aggressive disease, and ≥300 pg/μL as a “very high positive” marker strongly associated with systemic dissemination and poor prognosis.

Importantly, we validated the diagnostic threshold of 50 pg/μL against ROC analysis for all tumors combined. At this fixed cutoff, sensitivity was 82% and specificity 73%, compared to 95% sensitivity and 64% specificity at the ROC-derived optimal threshold (61.35 pg/μL) for all tumor types combined without a fixed threshold. This trade-off slightly reduced sensitivity but improved specificity supports the use of 50 pg/μL as a clinically practical baseline for initial screening, while higher thresholds remain valuable for risk stratification and prognostic assessment. By integrating ROC-derived evidence with clinically meaningful cut-offs, we provide a standardized framework for cfDNA interpretation that balances diagnostic accuracy with usability in diverse tumor contexts.

Additionally, our histological grade and stage–dependent ROC results (grade AUC = 0.83, sensitivity = 69% specificity = 95%; stage: AUC = 0.83, sensitivity = 72%, specificity = 82%) further support cfDNA as a prognostic biomarker. These findings are consistent with previous studies in canine oral malignant melanoma, which reported dynamic changes in cfDNA concentration and integrity indices throughout the clinical course, with marked alterations during metastasis or apparent tumor progression (29). Similarly, in canine lymphoma, (30) observed that 23 dogs with lymphoma had significantly higher cfDNA concentrations compared to 12 healthy controls, reinforcing the association between elevated cfDNA levels and disease severity.

To assess the early-stage predictive capabilities of cfDNA concentrations, we evaluated the ROC results obtained by comparing healthy versus low-grade tumor samples, while considering the limited statistical power associated with the small number of low-grade cases in our dataset (*N* = 22). The resulting AUC of 0.59, together with an optimal threshold of 31.1 pg/μL (sensitivity 64%, specificity 59%), and the performance metrics at this threshold (sensitivity = 0.59; 95% CI: 0.36–0.79, specificity = 0.64; 95% CI: 0.49–0.77; accuracy = 0.62), indicate that cfDNA concentration is more effective at distinguishing high-grade from low-grade tumors than at detecting early-stage disease. These findings support the interpretation that cfDNA functions primarily as a biomarker of tumor aggressiveness rather than an early detection marker.

This pattern is consistent with previous observations showing that cfDNA levels are substantially influenced by tumor burden and proliferative activity. Early-stage cancers often release extremely low fractions of tumor-derived cfDNA, whereas high-grade or advanced tumors shed significantly larger amounts, thereby improving detectability (31).

Our survival analysis provides compelling evidence that cfDNA concentration is not only a diagnostic marker but also a strong prognostic indicator in canine oncology. The significant survival differences observed across cfDNA ranges, particularly the sharp decline in the >300 pg/μL subgroup, suggest a link between elevated cfDNA levels and aggressive disease biology. The Cox model results reinforce this interpretation: while intermediate cfDNA levels (50–100 pg/μL) did not significantly impact survival, dogs with cfDNA between 100 and 300 pg/μL > 300 pg/μL had a substantially increased risk of death and those exceeding 300 pg/μL showed the strongest association with mortality. The global log-rank *p*-value (*p* = 2.68 × 10^−5^), and concordance index (0.74) indicate that cfDNA concentration is a meaningful predictor of survival outcomes. These results, aligned with previous studies reporting that elevated cfDNA levels correlate with tumor burden and poor prognosis in dogs. The lack of a significant difference between the lowest and intermediate cfDNA group reinforces the notion that higher cfDNA concentrations reflect increased tumor burden, proliferative activity, and cell turnover, all of which contribute to poorer survival outcomes.

Taken together, our data, contributes to a growing body of literature that positions cfDNA-based liquid biopsy as a viable non-invasive tool for screening and early detection, diagnostic support, and prognosis, offering high specificity and sensitivity that complements standard histological and imaging approaches. Incorporating cfDNA thresholds into clinical decision-making could enable earlier intervention and more tailored therapeutic strategies, particularly for dogs presenting with cfDNA concentrations above 100 pg/μL.

Several limitations warrant consideration. First, the sample size for tumor subtypes was limited, which may affect the generalizability of our findings. Second, cfDNA concentration can be influenced by non-neoplastic conditions such as inflammation or trauma, potentially confounding interpretation. Third, survival analysis was restricted to four tumor types, narrowing the scope of prognostic conclusions. For these reasons, we regard this work as a pilot’s study.

To strengthen the clinical utility of cfDNA-based stratification, future research should validate the proposed thresholds in larger, multi-center cohorts. Incorporating genomic profiling could further enhance specificity and refine diagnostic capabilities. Longitudinal sampling will be essential to characterize cfDNA dynamics during treatment and relapse, positioning liquid biopsy as a valuable tool in personalized veterinary oncology. In addition, integrating next-generation sequencing is expected to improve both diagnostic accuracy and prognostic power, advancing the precision and applicability of the OncoCan assay.

Finally, an important limitation in veterinary medicine is the difficulty of collecting sufficient blood volume, animals often move during sampling, resulting in smaller volumes and increased risk of hemolysis, which releases genomic DNA into plasma. In contrast, vacutainer systems used in human medicine, such as BD Vacutainer® or Streck cfDNA BCT tubes, employ closed-system collection, which minimizes hemolysis and prevents cellular genomic DNA contamination of plasma. These tubes stabilize cfDNA and reduce leukocyte lysis, which is essential for obtaining reliable cfDNA measurements. This difference highlights a key challenge when translating plasma cfDNA assays from human diagnostics to veterinary applications (32).

In this study, we also demonstrate that with the OncoCan test, it is possible to measure cfDNA concentration in a small volume of plasma of 2 mL obtained from just 5 mL of blood sample, which is an easy quantity of blood to collect in veterinary routine sampling, [OncoK9™ test (see text footnote 1) for instance required a minimum fill of 12–14 mL of whole blood], by selecting the fragment size range of 50–700 bp, which enables evaluation of degraded genomic DNA contamination based on the percentage of cfDNA fragments present in the sample.

## 5 Conclusion

Stratifying dogs by cfDNA concentration provides valuable insights for diagnosis, staging, and disease monitoring. Elevated cfDNA levels were associated with higher histological grades and disseminated disease, highlighting their potential utility in prognosis and therapeutic decision-making. The thresholds identified in this study may support earlier clinical intervention and enable noninvasive, longitudinal monitoring. Nonetheless, the observed variability in cfDNA profiles across tumor types emphasizes the importance of establishing tumor-specific reference ranges. Looking ahead, integrating next-generation sequencing (NGS) into the OncoCan assay will enhance its ability to detect neoplastic positivity and refine tumor classification, further advancing its role in precision veterinary oncology.

## Data Availability

The original contributions presented in the study are included in the article/Supplementary material, further inquiries can be directed to the corresponding author.

## Footnotes

1
https://antechdiagnostics.co.uk/oncok9/
