# Correction: OncoCan: a liquid biopsy assay for cell-free DNA quantification in canine plasma to support cancer prognosis

**DOI:** 10.3389/fvets.2026.1826236

**Published:** 2026-04-17

**Authors:** Virginia Sánchez, Gal.la Farreny, Elena Martinez-Merlo, Sara Trancon, José Carlos Montañés, Laura Enriquez, Arantxa Aguirrebeña, Agustín Arasanz Duque, Antonia Noce

**Affiliations:** 1 Complutense Veterinary Teaching Hospital and Department of Animal Medicine and Surgery, Veterinary Medicine School, Complutense University of Madrid, Madrid, Spain; 2 Clinica Veterinaria Alameda, Madrid, Spain; 3 Omica Biomed, Barcelona, Spain; 4 Molecular Biology Core, Hospital Clínic of Barcelona, Barcelona, Spain

**Keywords:** cancer, cfDNA, dogs, liquid biopsy, prognosis

In the published article, the authors' correct name and surname order was not followed in the authors list, which affected the Author contribution section, the citation, the copyright statement, and the Conflict of Interest statement.

The corrected authors' names in the authors list are:

Virginia Sánchez, Gal.la Farreny, Elena Martinez-Merlo, Sara Trancon, José Carlos Montañés, Laura Enriquez, Arantxa Aguirrebeña, Agustín Arasanz Duque and Antonia Noce

In the published article, the authors initials in the Author contributions section were incorrect. The statement was incorrectly written as “SV: Data curation, Resources, Writing – review & editing. FG: Data curation, Investigation, Methodology, Writing – review & editing. M-ME: Conceptualization, Data curation, Resources, Supervision, Writing – review & editing. TS: Writing – review & editing, Resources. MC: Formal analysis, Software, Visualization, Writing – review & editing. EL: Data curation, Methodology, Writing – review & editing. AgA: Conceptualization, Data curation, Resources, Writing – review & editing. ArA: Conceptualization, Data curation, Funding acquisition, Project administration, Supervision, Validation, Writing – original draft, Writing – review & editing. NA: Conceptualization, Formal analysis, Methodology, Software, Visualization, Writing – original draft, Writing – review & editing.”

The correct statement is:

VS: Data curation, Resources, Writing – review & editing. GF: Data curation, Investigation, Methodology, Writing – review & editing. EM-M: Conceptualization, Data curation, Resources, Supervision, Writing – review & editing. ST: Writing – review & editing, Resources. JM: Formal analysis, Software, Visualization, Writing – review & editing. LE: Data curation, Methodology, Writing – review & editing. AA: Conceptualization, Data curation, Resources, Writing – review & editing. AAD: Conceptualization, Data curation, Funding acquisition, Project administration, Supervision, Validation, Writing – original draft, Writing – review & editing. AN: Conceptualization, Formal analysis, Methodology, Software, Visualization, Writing – original draft, Writing – review & editing.

In the published article, the citation was incorrect. The citation was incorrectly written as “Virginia S, Gal.la F, Elena M-M, Sara T, Carlos MJ, Laura E, Arantxa A, Agustín AD and Antonia N (2026) OncoCan: a liquid biopsy assay for cell-free DNA quantification in canine plasma to support cancer prognosis. *Front. Vet. Sci*. 13:1768078. doi: 10.3389/fvets.2026.1768078”.

The correct citation reads “Sánchez V, Farreny G, Martinez-Merlo E, Trancon S, Montañés JC, Enriquez L, Aguirrebeña A, Arasanz Duque A and Noce A (2026) OncoCan: a liquid biopsy assay for cell-free DNA quantification in canine plasma to support cancer prognosis. Front. Vet. Sci. 13:1768078. doi: 10.3389/fvets.2026.1768078”.

The copyright statement for this article was incorrectly written as “© 2026 Virginia, Gal.la, Elena, Sara, Carlos, Laura, Arantxa, Agustín and Antonia. This is an open-access article distributed under the terms of the Creative Commons Attribution License (CC BY). The use, distribution or reproduction in other forums is permitted, provided the original author(s) and the copyright owner(s) are credited and that the original publication in this journal is cited, in accordance with accepted academic practice. No use, distribution or reproduction is permitted which does not comply with these terms.”

The correct statement is “Copyright ©2026 Sánchez, Farreny, Martinez-Merlo, Trancon, Montañés, Enriquez, Aguirrebeña, Arasanz Duque and Noce. This is an open-access article distributed under the terms of the Creative Commons Attribution License (CC BY). The use, distribution or reproduction in other forums is permitted, provided the original author(s) and the copyright owner(s) are credited and that the original publication in this journal is cited, in accordance with accepted academic practice. No use, distribution or reproduction is permitted which does not comply with these terms.”

The conflict of interest statement was erroneously given as “FG, EL, and NA are employees of Omicabiomed. AgA is a co-founder of Omicabiomed, and ArA is the CEO of Omicabiomed. The OncoCan test described in this study was developed by Omicabiomed. The remaining author(s) declared that this work was conducted in the absence of any commercial or financial relationships that could be construed as a potential conflict of interest.”

The correct conflict of interest statement is “GF, LE, and AN are employees of Omica Biomed. AA is a co-founder of Omica Biomed, and AAD is the CEO of Omica Biomed. The OncoCan test described in this study was developed by Omica Biomed. The remaining author(s) declared that this work was conducted in the absence of any commercial or financial relationships that could be construed as a potential conflict of interest.”

The original version of this article has been updated.

